# The Helpfulness of Spiritually Influenced Group Work in Developing Self-Awareness and Self-Esteem: A Preliminary Investigation

**DOI:** 10.1100/tsw.2005.99

**Published:** 2005-09-28

**Authors:** Diana Coholic

**Affiliations:** Laurentian University School of Social Work, Sudbury, Ontario, Canada

**Keywords:** substance abuse, group work, social work, spirituality, experiential, Canada

## Abstract

This paper discusses an exploratory study that investigated the helpfulness of spiritually influenced group work with eight adult women who shared a history of substance abuse. The overall purpose of the group was to help participants develop their self-awareness and self-esteem. The group, which was contextualized in transpersonal theory, was organized around the following themes and experiential exercises: meditation, mindfulness practice, dream work, stream of consciousness writing, the shadow self, and other arts-based processes. Grounded-theory analysis of group sessions and individual interviews with the participants found that the participants perceived the group to be helpful in developing their self-awareness and self-esteem. While the participants identified different aspects of the group as spiritual, making-meaning was one practice that was consistently described as a spiritually sensitive process. The results of this study in this emergent field are promising and suggestions are provided for future research.

### INTRODUCTION

A transition is occurring within the scientific and academic community in that a spiritual dimension is increasingly being considered[1,2]. Indeed, researchers at major universities in North America are studying connections between the mind, body, and spirit[3,4,5,6,7]. Within social work, there has been a burgeoning development of literature (particularly in the past decade) that discusses the incorporation of spirituality in helping[8,9,10,11]. This literature generally refers to spirituality as a universal aspect of human life that encompasses experiences that transcend the self; it is a feature of human life and development that is accessible to all. The rationales for incorporating spirituality into social work practice include: issues raised by clients, cultural diversities, coping resources and resiliency factors, social work's holistic approach, the need for knowledge development, and the effectiveness of spiritually influenced interventions[12,13,14,15,16,17,18,19,20,21,22,23,24,25,26,27,28,29].

We are especially interested in the latter idea that spiritually influenced practice is effective. Presently, this belief is evidenced in the literature by way of case studies, survey research, practice wisdom, and personal experiences[30,31,32]. Although there is great merit in practice wisdom and in constructing knowledge from practice, especially when considering an experiential construct such as spirituality, the accuracy of these beliefs must be explored. Social workers have not yet investigated whether attending to spirituality actually improves client outcomes or not[1]. At the same time, the belief in spirituality's helpfulness underpins many of the arguments for the inclusion of spirituality in practice and education. For example, it has been contended that when spirituality is ignored, the fulfillment of people's potential for wholeness, their right to fully express their experience, and creative transformation are all restricted[33,34]. Processes such as meditation, prayer, and ritual have all been described as effective[12,33]. Also, practitioners have described the more rapid progress of clients when spirituality was addressed, and even social work students with little experience or knowledge of the social work and spirituality literature believed in spirituality's effectiveness[35,36]. Studies that aim to learn more about how spiritually influenced helping occurs and that examine its helpfulness constitute one of the next important steps in the development of this body of knowledge, and it is these goals that our research has attempted to explore.

Consequently, this paper describes a pilot study that investigated the helpfulness of a spiritually influenced group program that had as its overall goal the improvement of participants' self-esteem by way of developing their self-awareness. Trying to improve people's self-esteem and self-awareness is a basic aspect of most helping approaches[37], and a high level of self-esteem is linked with resiliency and the ability to cope[38]; they are key elements underpinning healthy human development[39]. The uniqueness of our group program is its focus on spiritually influenced practices, and the connections between these practices and the development of self-awareness and self-esteem. Specifically, the paper discusses what the participants found helpful and spiritual about the group. For instance, we found that the group participants experienced different practices as spiritual except for the process of making-meaning, which they all expressed to be linked with their spiritual beliefs. Other group processes that were identified as helpful and as spiritual experiences were dream-work, meditations and mindfulness practice, and feelings of connectedness among group members.

### AN OVERVIEW OF THE STUDY AND GROUP PROGRAM

The pilot study was completed in May 2004, with the support of the Northern Regional Recovery Continuum (NRRC), a community agency that works with women and addictions (see www.lakesidecentre.ca). The group program consisted of six sessions in total, each of which was 2 h in length. The group sessions utilized various experiential and arts-based exercises that are integral in other spiritually sensitive approaches for the purposes of building self-awareness and self-esteem such as stream of consciousness writing, relaxation, walking meditation and mindfulness practice, and dream-work[29,40,41]. Initially, the group was facilitated with ten participants affiliated with the NRRC, but one woman dropped out of the group for reasons unknown to us, while another woman was asked to leave by the NRRC due to a relapse — thus we ended the group with eight participants. The study explored how spirituality is discussed in group practice and experienced by group participants, examined the group participants' perceptions about the group processes and its helpfulness (or not), and collected self-reports of self-esteem before and after participation in the group as another measure of the group's influence or perceived helpfulness (or lack thereof). We used the Multidimensional Self-Esteem Inventory to collect these self-reports[42].

Transpersonal theory offered a useful theoretical base for the development of this group, as there are very few psychological or social work theories that specifically address the spiritual dimension in life. Transpersonal theory offers a way of making sense of and discussing spiritually influenced practice. Transpersonal simply means beyond the person or ego — there is a higher or inner self distinct from the personal ego, for example, transpersonal approaches focus on an expanded theory of human consciousness. Transpersonal theory builds on the work of Jung, Assagioli, Maslow, Rogers, Wilber, Walsh, Vaughn, and others[43]. It has been called the "fourth force" in psychology because it challenges and moves beyond the theoretical influence of the first three movements: psychoanalytic, behaviorism, and humanistic psychology[44]. In general, transpersonal theorists advocate for methodological pluralism and multidisciplinary helping methods, a cross-cultural approach to building knowledge, a belief that spiritual experience is universal, and that an appropriate stance is one that includes a component of curiosity and openness to mystery. In terms of practice, transpersonal theory is described as a modality that seeks to establish a growth-producing link between a person and transpersonal experience, and has an interest in the spiritual, which includes intuition, meditation, relaxation, visualization, and dream-work[44]. Canda and Smith[45] pointed out that transpersonal theory has only recently begun to influence mainstream social work although it has been growing steadily within psychology and philosophy since the 1960s. In particular, Nexus, Transpersonal Approach to Groups was a good source of information for us in developing the group program[40].

The six group sessions were organized around the following themes: an introduction to the importance of process and the nature of the group work, stream of consciousness writing, the shadow self, mindfulness meditation, dream analysis, and ending and evaluating the group experience. Session one was an introduction to the group. The nature of the group was explained to the participants and exercises were used to help establish a context in which participants would feel comfortable with their diversity, and learn to focus on the importance of the process of learning about themselves and improving their self-esteem. A short introductory relaxation/meditative exercise was conducted (meditations were used in every session). Discussion also occurred regarding their feelings about creativity and art. At the end of every session, books were made available to the participants to borrow[46,47,48,49,50,51,52].

In session two, the facilitator explained stream of consciousness writing. The goal of stream of consciousness writing is to become more aware of the constant dialogue in our minds, that is, the process helps us to connect with inner thoughts and feelings, strengthening self-awareness. Importantly, it was stressed that there is no evaluation attached to the writing and that judgments should not be made about the process especially while engaged with it. Clay work followed the writing exercises. One participant explained that if she had not engaged in the stream of consciousness writing first, she would not have been able to sculpt a beautiful flower. She explained:

> "Because by writing this down and just letting our minds flow and stuff, I ended up in a positive space and then I made my flower…it's like I brought myself inside and it stayed there and then I made my stuff. Because if I would have done this just before, then I wouldn't be focused on the positive and I was just feeling like, I was a mess you know…so I wouldn't have made it, anything pretty, I would have just made a flower…I wasn't feeling unique and individual, I was feeling lonely."

The shadow self was the theme in session three and it was discussed as encompassing those things that we hide from ourselves and the world such as feelings like shame and anger — essentially, qualities and feelings that are held in our unconscious because they are unacceptable to our conscious mind. The shadow is a term coined by Carl Jung[53,54]. Simply, the idea is that we need to acknowledge the parts of ourselves that we hide, deny, and repress in order to know and understand ourselves fully. However, the process of doing so is much more complex, because it involves developing self-awareness of aspects of ourselves that we would rather not acknowledge.

To begin session four, an exercise of mindful eating was conducted with small bits of food such as raisins and cereal pieces. Discussion included the idea that mindful moments help us to become aware of what is transpiring in our environment, of our inner workings, and our reactions to situations. The idea of interconnection was also raised, for example, contemplating all the people that it took to create one raisin. The participants also engaged in a walking mindfulness meditation outside at a running track located very close to where the group was being held.

Session five began with constructing dream collages. The participants were instructed to scan through the provided magazines and to extract anything that seemed associated with their dream images (a type of free association exercise). The facilitator explained that dreams are often multilayered and have meanings on many different levels, so the layering of the pictures in a collage is completely appropriate. As well, dreams use both language and images, which can be represented in the collage. Participants were encouraged to interpret symbols and images according to their own experiences and viewpoints — developing their own meaning was important. Group discussion and analysis of the dream collages was conducted.

The final group session began with a guided imagery exercise focused on interconnections. One of the group's goodbye rituals included discussion about both the challenges and highlights of the group, and their future goals.

### The Process of the Research Study

Potential participants were recruited from the NRRC (Northern Regional Recovery Continuum) using Participant Information Statements, which provided them with a brief description of the group program's structure and goals. After women indicated an interest in participating, they met individually with the principal investigator (PI) prior to beginning the group so that the goals of the group could be discussed, questions could be answered, the consent form signed, the demographic form completed, and The Multidimensional Self-Esteem Inventory (MSEI) administered. A research assistant (an experienced practitioner) assisted in developing the group and facilitated the group sessions. The PI and the facilitator met after each session to discuss the group process and to address any issues that arose. On completion of the group, the PI met individually with the eight group participants in order to conduct a semi-structured interview that explored their experiences of the group, and their viewpoints on the helpfulness (or not) of the process. This was also an opportunity to discuss the results of the MSEI, which was administered again at the end of the last group session. The group sessions and the postgroup individual interviews were all audio-recorded for the purposes of grounded theory analysis.

### Brief Description of the Research Participants

The participants ranged in age from 20–53 years. All had been previously involved with the NRRC either through the 21-day inpatient program or in aftercare groups. A few were still actively involved with the agency and lived in the agency's aftercare program and/or attended other agency groups. With regard to their spiritual and/or religious affiliations, one participant identified as First Nation (Aboriginal). Two identified as Catholic, another stated that she was raised Catholic and sometimes attended church. A fifth participant said that she was raised Catholic, but does not practice anymore. One participant reported that she was raised Baptist/Pentecostal and that she currently attended a Pentecostal church. One participant was raised Protestant. One woman was raised in the Mormon religion, but stated that she does not practice anymore. One participant stated that she was affiliated with the Rosicrucians, Billy Graham, and bible studies such as "A Course in Miracles". Finally, one participant reported that she was raised Catholic, but now attended a Pentecostal church.

### Research Design

While both quantitative and qualitative methods are utilized in practice-based research, qualitative methods such as grounded theory approaches were better suited for this study for several reasons. First, grounded theory methods are inductive approaches that aim to generate theory. This research was exploratory with the goal of discovering new knowledge because this is a developing field. Indeed, a widely accepted practice framework for spiritually sensitive work has yet to be developed. At this stage, we need to better understand how spiritually influenced practice actually transpires in the real world with real people and all the complexities they bring to the helping process. Second, practice professions such as social work and nursing have made considerable use of grounded theory methods in their quest to conduct research that is relevant for practice[55]. Indeed, the demand for solid links between practice and research has consistently been a strong theme in social work[56]. Grounded theory methods enable the discovery of knowledge from the world of practice, which in turn produces frameworks that are useful for practitioners and consumers. Third, a grounded theory approach allows an investigation of spiritually influenced practice with the ability to respond to unexpected events that often occur when conducting psychotherapy with people, that is, we can improvise to meet participant needs[57,58].

### Grounded Theory Methodology and Analysis

A grounded theory method helped us construct knowledge from the analysis of group sessions and research interviews with participants. Research data (transcribed group sessions and individual interviews) was converted into systematic schema for examining its meaning, discovering themes and patterns, and making connections among concepts — making links between themes is a means of putting conceptual order on the mass of data. Final integration of research data follows this process, but done at a higher, more abstract analytical level[59]. For example, all of the participant interviews were coded and sorted into relevant categories. As new data emerged, they were either clustered into already-existing categories saturating those groupings or, particularly in the preliminary stages of analysis, they were sorted into a new category. As the analysis continued, the categories were continuously compared with one another and with new emerging data in order to discover links between the groups. Any categories that are related are combined and once again compared to incoming data to assess their relationships to hypotheses that emerged from the comparisons among groupings. Refinement of the categories that have emerged from the analysis comes from the continued connection and reduction of concepts that leads to increased abstraction[60,61]. Grounded theory analysis allowed us to identify the intricacies of how spirituality is actually discussed in group practice, how it shapes practice processes, and how participants perceived the helpfulness of the spiritually influenced work.

### The Multidimensional Self-Esteem Inventory (MSEI)

The use of the MSEI in this study adds another layer of data collection and analysis of self-reports of self-esteem. The MSEI is an objective self-report inventory that provides measures of the multidimensional components of self-esteem[42]. Respondents complete the MSEI by entering their responses on a rating sheet: for section 1 they are directed to use a 5-point Likert scale to report how accurately the 61 items describe them, and for section 2 they are instructed to use a 5-point Likert scale to report how often they experience the thoughts and feelings described in the 55 items. The MSEI takes approximately 15–30 min to complete. In operationalizing theory into concrete measures, the MSEI uses the following 11 scales: global self-esteem, eight component scales (competence, lovability, likeability, self-control, personal power, moral self-approval, body appearance, and body functioning), identity integration as a measure of global self-concept, and defensive self-enhancement as a validity measure to provide information on the degree to which a person is defensively inflating her self-presentation. The MSEI has undergone extensive conceptual and psychometric development over a 10-year period[42].

## ANALYSIS AND DISCUSSION

The following discussion specifically focuses on how spirituality was discussed in the group and experienced by the participants, particularly as this relates to their development of self-awareness and self-esteem. The presentation of the research findings is meant to be an overview. It is beyond the scope of this paper to examine each topic, such as mindfulness, thoroughly. However, the discussion demonstrates how spirituality can enter into group helping processes and provides direction for future study in this emergent area. Although spirituality was deemed to be a really important part of the group, the participants experienced different aspects of the group as spiritually sensitive; this should be expected given the diversity of their backgrounds and the complex nature of spirituality. However, it can be noted that two of the participants reported that the entire group experience felt spiritual for them.

### Working with Dreams

Although many practitioners feel unprepared to attend to their clients' dreams, the usefulness of dream analysis is increasingly being considered across helping approaches and its connection with spirituality is evident in the literature[62]. Krippner et al.[63] explained how dreams are intimately connected with major religious and spiritual traditions throughout the world. France[40, p. 129] made the point that since the beginning of time, all cultures regarded dreams as having some special power that transcended the past, present, and future, and that many cultures believed that dream messages are the vehicle through which God can speak. In group work, dream exploration can allow participants to go beyond the physical world into the spiritual realm. Indeed, dream exploration constitutes an important part of the psychotherapeutic work in transpersonal approaches[40,44,53].

Along the same lines, for some of the group participants, dream-work was connected with spirituality. For example, one participant reported that a lot of her dreams guided her to action. She stated, "God was letting me know this all along, that my addiction had to quit, so I went into rehab after [having the dream]." One participant discussed how a recurring dream was actually representative of a past-life experience. She used the dream to make-meaning of her current life situation and family dynamics. Yet another participant explained that further reading about dreams elicited a spiritual experience. She described:

> It came to me [understanding]…it popped out of the book. That doesn't usually happen, so for me that's more of a spiritual thing because usually if I start reading, I'll ask my higher power to let me grasp what I really need to grasp. So that means…if I don't understand, it's not made for me to actually get it today.

In the individual interviews, six of the participants described how working with their dreams was helpful, while one woman stated that she had difficulty making connections between the dream collage and her life experiences. They also identified that there was not enough time to analyze their dream collages fully in the group. However, several women reported that they brought the collages home and continued to work with them either on their own or in individual counseling. One participant described her group highlight as constructing the dream collage. She found the analysis that occurred in group helpful to developing her understanding of the dream's meaning for her. We agree that self-analysis or the "dreamer as authority" is essential[64], and the relevance of the participants' self-analysis of their dreams was emphasized. Another participant explained that working with dreams increased her self-awareness by helping her connect with unconscious messages. Several women reported that they would continue to use dream-work for self-discovery and healing.

Similarly, within the research literature, dream-work has been reported to be useful. Moss[65] argued that dream-work can help people access deep unconscious feelings in order to facilitate a more complete mourning process. Barrett[66] stated that dreams represent a powerful metaphor for irrational beliefs that might not otherwise be articulated. In group counseling, the analysis of dreams has been reported to help with understanding other group members, promoting group cohesiveness, and stimulating therapeutic group interactions[67]. Even cognitive therapists have begun to examine the usefulness of dream-work and devoted a special issue of the *Journal of Cognitive Psychotherapy* to this topic [see Vol. 16(1), 2002]. Dream analysis and the other exercises utilized in the group do not have to be classified as spiritual experiences. However, as is evident in the participants' comments above, these processes make sense to some people as spiritual experiences, and the discussion of dreams may naturally lead to considering spiritual viewpoints. If helping practitioners remain uncomfortable with considering the spiritual dimension of people's lives, they may miss the opportunity to help the client construct a holistic narrative that best fits their experience.

### Meditations and Mindfulness Practice

The facilitator introduced the participants to a variety of meditative exercises in order to address different preferences. Each session began with a meditation or meditative exercises were incorporated later in the session in order to help ground and connect the participants to the work of the group. Meditating before engaging in experiential exercises and other psychotherapeutic work can help to quiet the mind and open up the possibility for a stronger connection with feelings and/or unconscious processes. Self-awareness and insight arise from an ability to pay attention to and experience one's anxieties, fears, and other feelings that often reside in the unconscious mind. Frattaroli[68, p. 194] contended that you can think of this process as simply getting in touch with a feeling or, more profoundly, as listening to the soul.

For some participants, the meditations felt spiritual because they were taking the time to connect with my [higher] self, while for others it was an opportunity to feel their energy and other people's energy, or to connect with their higher power. One woman believed that the meditations allowed conversations about participants' higher powers and spirituality because everybody had something to contribute. On the other hand, one participant explained that while the meditations were relaxing, they did not feel spiritual to her because they were not long enough or in-depth enough. This participant experienced the stream of consciousness writing as the most spiritual exercise, because for her, it was directed by God. She stated, "…it continued to flow and I know it was from within me and it wasn't me. And how God, in my writing, came out."

For some of the participants, the meditations were difficult at first because this was a new experience for them, and one's ability to meditate and relax generally becomes easier and more effective with practice[41]. However, several participants discussed how the meditative exercises were particularly helpful in developing their self-awareness. One woman explained how through the use of meditation and guided imagery (with the help of her sponsor) she was able to visualize rescuing her "inner child" and so felt for the first time in 20 years that she could protect herself from abuse. She stated saying that meditation is a gift, "…is like wow, it really is and I just can't get over what I've accomplished, what I've achieved through meditation…I found my heart…It's like I found my soul-mate, and it's in me!" Another participant also explained how learning meditation has helped her to feel less congested than normal with thoughts racing through her mind. Some women stated that they liked meditating at night because it helped them sleep, while others preferred to do it in the morning because it helped them deal with the day ahead.

Mindfulness practice is a specific form of meditation. Jon Kabat-Zinn explained that the key to mindfulness meditation is an appreciation for the present moment and the cultivation of an intimate relationship with it through a continual attending to it with care and discernment. While mindfulness lies at the root of Buddhism, Taoism, and yoga, it is also found in the works of Emerson, Thoreau, and Whitman, and in Native American wisdom[69]. Kabat-Zinn further proposed that an operational working definition is: The awareness that emerges through paying attention on purpose, in the present moment, and nonjudgmentally to the unfolding of experience moment by moment[70]. Recently, mindfulness has been adopted as an approach in contemporary psychology for increasing awareness and responding skillfully to mental processes that contribute to emotional distress and maladaptive behavior[71]. Indeed, mindfulness practice is increasingly being studied in various fields with a myriad of client populations and problems, with research results indicating its effectiveness. For example, researchers have found that mindfulness is effective in helping chronic pain patients deal with grief[72], in helping a broad range of individuals cope with problems[73,74,75], in fostering health benefits[74,75,76], in decreasing mood disturbance and stress[77], in treating negative body images[78], and it has been proposed as a core common factor in psychotherapy[79], to name just a few examples. In harmony with these results, most of the group participants reported that learning mindfulness was helpful in assisting them to better appreciate life's moments and themselves, and in understanding themselves more fully.

For example, one participant reported that the mindful eating exercise helped her to actually taste the food, which was a different experience for her "because I don't really taste my food, really". Another participant discovered that normally she would have swallowed a handful of the raisins before learning that she really thought they were gross. This was affirmed by another participant who stated that "she thought I liked the little squares…and I started tasting it and you were right, I don't really like them". The mindful walking exercise was also a new experience for most of the participants. One woman reported her new-found awareness:

> "The awareness is great, when it overcomes you, when you become very aware how big the world is. I know that sounds weird but I'm thinking of myself and feeling like my body walking, and then I'm seeing everything around me and it's like "wow, it's just me here walking on this huge big world."

Three participants identified that learning about and being more mindful facilitated feelings of gratefulness. One woman explained that she can now appreciate more the small details that we all take for granted. Importantly, she reported "that it's making things more worthwhile…it fills my day with healthy things…I don't feel like I'm wasting my time," which in turn helped her to feel better about herself. Another participant summed up her experience by reporting that mindfulness has encouraged her to feel happier, calmer, and more confident. For her, the mindfulness practice was "the last of the steps that I needed…the program that we're putting together here, what I'm planning incorporating in my life, it seems to be just the thing to complete it [her healing]." Mindfulness is a good example of a helpful process that is rooted in spirituality, but that can be learned by many people with both diverse spiritual beliefs or even no spiritual standpoints. Perhaps this accounts, in part, for the current interest in mindfulness across helping approaches and disciplines. However, we should contemplate the complexities involved in divorcing mindfulness from its spiritual roots. As Kabat-Zinn[70, p. 145] argued, it is important that we recognize the unique qualities of mindfulness meditation practice so that it is not "simply seized upon as the next promising cognitive behavioral technique or exercise, decontextualized, and plugged into a behaviorist paradigm with the aim of driving desirable change, or of fixing what is broken."

### Connectedness

The idea of connectedness and helping to foster connections is a concept found in the spirituality and social work literature; it has been identified as a spiritually influenced helping process. For example, feminist social workers have discussed the process of helping clients to foster connections with community, in groups, and with their feelings as spiritually influenced practice. They have also described the spiritual connections they sometimes form with clients[80]. Ballou[18] argued that feminist spirituality is grounded in community, connection, and relationship, while Kimmel and Kazanis[25] described the deep spiritual connections that can occur in counseling groups. These "deep connections" can be conceptualized as spirit-spirit connections. Zukav[81] described this by arguing that we are all souls wearing Earth suits (our gender, color, etc), and that people are increasingly coming to recognize each other as a fellow soul who is part of a universal family, rather than focusing on the particular Earth suit they have donned for this lifetime.

The connectedness experienced between the group members was discussed as feeling spiritual and a spiritual experience by some of the participants. For example, one participant stated that "just the way everybody…got so involved…it felt (pause) it was really good…I found everyone was at ease…more relaxed and at peace in that group than a lot of the other groups you can go into." Another woman found it hard to put into words her experience of this connectedness, but said that the group was about being at peace "…Like nobody judged what you said…And now I see how much confidence everybody had in one another." For another participant, the experience of spiritual connection felt inspiring and encouraging even after the group was completed. Articulating experiences that are based in feeling and that feel spiritual can be difficult. We lack the language to describe and capture these processes fully. However, based on these and other experiences reported to us by the group participants, it would seem that a spiritual connectedness among the participants accounted, in part, for the group's camaraderie and respect for one other, which was evidenced throughout all of the group sessions.

### The Importance of Making-Meaning

The process of making-meaning has been identified in the literature as a practice principle for spiritually sensitive practice, and has been described as a spiritual practice because spirituality itself is often defined as a process of making-meaning[21]. Barrett[19] described healing from trauma as a quest for spirituality that reflects a deep need for meaning. Anderson[82] stated that spirituality is the experience of making-meaning informed by a relationship with the Transcendent in life. Thus, therapy from a spiritual perspective aims to help people fashion narratives that weave together human and Divine realities, enabling people to hear their own stories retold with clarity and new possibilities, transforming their lives in the process.

Certainly for the participants in this group, spirituality helped them make-meaning of their struggles with addiction. In the first group session, one participant stated that she always "felt I was here for a reason…I definitely do believe I'm here at this moment for a reason." Another woman agreed and discussed her belief that people have different troubles in their lives for certain reasons that they need to figure out. She stated:

> "I know about my disease but now I have to find what else I was put on this earth [for]…right now it's to learn about myself…this is one of the processes of life, a journey that we have to attend to. That's the way I see it in a spiritual way."

Similarly, a third participant explained that the path she is on was meant for her, even though some of it has been bad. She stated that "…everything I've done in my past makes me a better person for today and will help me in the future." Another participant stated that she agreed that everyone has a purpose, although she was not sure who made up that purpose. She raised the question: Is it was possible to reach a spiritual peak in life" In response to this query, another participant reported that if she does not finish her life journey the right way that she's going to come back [in another life-time]. Yet another woman asserted that "no one reaches their spiritual limit until they die and go on to the spiritual world…we're on a journey and we have to go through these experiences for us to look at ourselves…I don't believe in coincidences…because our paths were meant to cross."

These are just a few examples of how the process of making-meaning feels spiritual to some people or gets interpreted in a spiritual way. Making-meaning is the one process that stands out in the analysis because it was discussed in all of the group sessions. Also, it entered into the discourse on different layers. For example, as noted above, spirituality permeated the participants' more global understandings of why they had problems and what they needed to do to heal. But spirituality also helped them to make sense of the specific exercises and processes they engaged with during the group. As the participants noted, God or their higher power communicated with them via their writing, reading, and dreams. The participants' comments illustrate the importance of making-meaning of experiences as part of the process of healing, integrating one's life experiences, and continuing to grow and develop as a person. Because assisting people to make sense of or make-meaning of their life events is such a basic element of many helping perspectives, practitioners should be open to integrating people's spiritual viewpoints into this process. When we create the space or grant permission for spirituality to enter into peoples' stories, they will raise it and incorporate it when required. We can then engage in meaningful and helpful discussion with them in order to cocreate more complete and healthy life narratives that make sense for them.

### Self-Esteem Measures

We analyzed the test scores from the MSEI as follows: First the data were tested for normality. Because none of the data were normal (they were more evenly distributed across a range, which is not unusual for these types of data), we used paired Wilcoxon tests (a nonparametric test that is the equivalent of a t-test for non-normal data) on all 11 scales for pre- and postgroup scores. For each scale, the postgroup scores were significantly higher. All of the scores were at least $p < 0.05$. Due to uncontrolled variables, we cannot assume that this increase in self-esteem scores can be attributed solely to the women's participation in the group program. However, it was still a useful process to discuss the improvement in scores with the participants in the individual interviews. They were pleased with the documented increase in scores and the areas in which they continued to score low provided material for their consideration. Interestingly, the participants attributed their increase in self-esteem scores in part to their participation in the group and their increased selfawareness. For example one participant stated: "I'm feeling more positive now than before. I put everything I had into the group, like everything that was required of me to do, I did the best that I could…like this really helped plus I've taken a course on Monday for relapse prevention. So between the two of them…it's really keeping me focused and on track."

A second participant said that "the group helped me to deal with issues…I'm now able to sit down with myself through meditation or whatever…I can just focus on stuff that I've learned…I've achieved something…I've grown so that I don't have to feel less than." A third participant explained that she would attribute over half of the change in her self-esteem to the group with the rest of the change coming from engagement with individual counseling. A fourth participant who reported that the group processes were right up her alley reported that she feels better about herself in that she is surer about herself and what she needs to do to keep developing; her expectations of herself are much more realistic now and positive. She argued that 70–75% of her change was due to her group participation. A fifth participant spoke about how the spirituality of the group and specifically the dream-work was important in developing her self-esteem. She explained that the change in her scores was pretty much all about the group because the group helped her to amalgamate all of her previous learning and further develop her healing — to actually go there and [resolve previously identified issues]. A sixth participant reported that the group helped her to develop her self-esteem by encouraging her to identify her strengths and confidence in her own abilities. A seventh participant explained that the group helped develop her self-esteem by "putting balance into my life. Also, having fun through recovery makes you even more open in sharing because it doesn't have to hurt this time. I can sit here and laugh and I'm still being open. So I guess it teaches us another level of intimacy." Finally, the eighth participant explained how the creativity of the group was relaxing and life-changing. She was able to become more creative at home with her children so that they saw a part of their mother that they had not previously experienced. She reconnected with her children on their level, and "the kids, I think, noticed something different…I mean the kids are my life, so that's really had an effect."

Thus, for some participants, the group was a profound experience in itself that was significant in helping them to develop their self-awareness and self-esteem, while for others it was a helpful adjunct to other strategies for change such as individual and other group counseling.

### The Group's Purpose and the Idea of "Fun"

One final point can be made concerning the nature of the group. We did not realize how different this group would be for most of the participants. Although a few did have previous experience with meditation and experiential therapeutic tools such as journaling, for most of the women, the techniques used in the group constituted a very different experience particularly in relation to building self-awareness and self-esteem. Six of the participants discussed the group difference in the individual interviews. One woman stated "that she found it really different. Just the hands on…at first I wasn't sure about the group because to me it felt like a little kid playing with play-dough." Another participant explained "that the group program was different in that we didn't have to be as deep and talk deeply [about]…our sobriety and things. And we didn't have to cry." Similarly, another participant stated "that it was a very different group…I was thinking, well I'm not getting anywhere with this group because I didn't melt-down today, I didn't cry today…because we were having fun through it all. And I think that's something that I know that I was missing and that all women were saying that they were missing. Because ever since getting into recovery everything has been so serious, so intense…it doesn't have to hurt, at the moment, for you to be able to grow."

Along these lines, another participant described the group as "refreshing...I don't know how else to explain it, because it felt good. It was fun…and we laughed a lot I think." One other participant discussed how this group fostered balance in her overall process of recovery. The concept that the participants were having fun while they were learning about themselves and new ways of addressing issues without feeling like they had to "pull out your wisdom tooth or something" is interesting, as it can help us expand our notions of the helping process as including both pain-filled experiences with other more joyful processes. Another woman summarized this sentiment well: "I was longing for a group where I [could] deal with my issues in a positive way…I think in the first two sessions I was thinking…if I'm not sitting there and having a hard time and crying, I am not dealing with anything…But this group…allowed me that connection with my inner child…it's brought up a lot of past issues, stuff that I didn't want to remember but gave me the opportunity to deal with it."

The principal investigator did meet with each participant before the beginning of the group to explain the nature and purpose of the group, but this was clearly not enough. In the future, we will spend more time in the first session building an understanding of how experiential techniques and processes can be helpful in developing self-awareness and self-esteem. Otherwise there could be a risk that people will drop out or not utilize the group experiences to their fullest potential.

### CONCLUSIONS

This paper discussed how spiritually influenced group work was useful in helping participants strengthen their self-awareness and self-esteem. One of the goals of this study was to learn more about how spirituality is actually discussed in group practice and how it can shape the helping process. It was evident in the first session that the participants felt comfortable raising their spiritual viewpoints, which affirms for us the importance of making room for the discussion of spirituality and the willingness of people to raise it, when this space is created. Because the participants were informed that the group included the spiritual dimension in their self-discovery process, discussions that took place naturally involved this facet of people's lives. One aspect of how the participants discussed spirituality stands out in this group. For instance, most of the participants talked about their spiritual beliefs as a way of helping them to make-meaning of their life circumstances. This is interesting to contemplate with regard to social work and other helping perspectives, because we are often involved in helping people to make sense of life events. For this group of women, spirituality was an integral part of this process.

One can make room for spirituality to enter into healing spaces, but participants will find and experience spirituality differently. For instance, for a couple of the participants, the whole group felt spiritual. For other women the meditations were the most spiritual experience, while for others the dream-work and/or mindfulness elicited spiritual connections. So, for some people, meditation is a chance to connect with a higher power, while for others it is simply a means to becoming grounded, calm, and focused. The important point here is that in a group that incorporates the spiritual realm, participants are encouraged to bring their spirituality to the process and to make sense of their experiences in a holistic manner if they so wish and if they deem this to be important for them. And for some people, spirituality is how they make sense of a particular process; for example, one participant described stream of consciousness writing as a way that God can speak to her. While the experiential exercises conducted in this group do not have to be classified as spiritually influenced practices, we should keep in mind that they are experienced as spiritual experiences for some people.

We were also interested in learning more about the helpfulness (or not) of incorporating spirituality into group practice. According to the participants, the group was helpful in a myriad of ways including helping to create a shift in self-esteem. The participants found that the meditations assisted them to become more self-aware by helping them to visualize therapeutic processes, or by helping them connect more strongly with their thoughts and feelings by providing focus. Mindfulness practice helped some women change their self-perceptions and fostered feelings of gratefulness. They learned that healing can be "fun" and creative, and that creativity could help them build relationships with their children, others, and themselves. For some, the group was the missing link in their recovery. For others it enabled them to expand their healing to another level or helped them to understand and amalgamate previous work they had completed. The group also served as a useful adjunct to other therapeutic work they were already engaged in.

There is much more work required in continuing to build knowledge about spiritually influenced helping. It can be noted that the group goals and its processes are generic enough to be applicable with various clientele. It will be important to study how spiritually influenced practice works with different client populations and problems because social work practice is so diverse. Also, as knowledge continues to be built in this field, future studies may explore the helpfulness of spiritually oriented practice compared with other approaches and control groups. The possibility of bringing people from different backgrounds together to discuss spirituality was also reaffirmed. However, this is something that future studies may want to explore in greater depth. Although the women in our group held different belief systems, the differences were not as radical as they could be. Specific processes such as dream-work and mindfulness, two exercises identified as particularly helpful in this study, could also be investigated further. Although several of the participants stated that they used the group exercises outside of group, we do not know how long this learning and practice was sustained. Also, it would be interesting to conduct a spiritually influenced group with participants who have knowledge and more experience of some of the processes used in group: How would their experiences differ? In conclusion, the results of this current study are promising in terms of learning about how spiritually influenced group practice takes place and is experienced as helpful, and they provide impetus for future study in this emergent field.

### ACKNOWLEDGMENTS

The Northern Regional Recovery Continuum funded and supported this study. I would like to acknowledge the work of Julie LeBreton, BSW who helped to develop and facilitate the group program.

### BIOSKETCH

**Diana Coholic**, **MSW, PhD, RSW** (registered social worker), Assistant Professor, Laurentian University School of Social Work, Ramsey Lake Road, Sudbury, Ontario, Canada, P3E 2C6. Tel: 705-675-1151, ext. 5053/Fax: 705-671-3832. E-mail: dcoholic@laurentian.ca
